# Molecular determinants of *Escherichia coli* causing neonatal invasive infection following vertical transmission

**DOI:** 10.3389/fcimb.2026.1855839

**Published:** 2026-06-15

**Authors:** Liang Gao, Jia yin Wu, Xue rong Huang, Xue yan Lin, Dan tong Lin, Yao Zhu, Xin zhu Lin, Ji dong Lai

**Affiliations:** 1 Department of Neonatology, Department of Pediatrics, Women and Children’s Hospital, School of Medicine, Xiamen University, Xiamen, Fujian, China; 2 Xiamen Key Laboratory of Perinatal-Neonatal Infection, Xiamen, Fujian, China; 3 Department of Clinical Laboratory, Women and Children’s Hospital, School of Medicine, Xiamen University, Xiamen, Fujian, China; 4 Department of Obstetrics, Women and Children’s Hospital, School of Medicine, Xiamen University, Xiamen, Fujian, China

**Keywords:** clonal virulence complex, *Escherichia coli*, K1 capsule, neonatal invasive infection, risk stratification, ST95, vertical transmission

## Abstract

**Background:**

*Escherichia coli (E. coli)* is a leading cause of life-threatening neonatal invasive infections via vertical transmission. However, the bacterial molecular features that distinguish invasive infection from asymptomatic colonization following vertical transmission remain unclear in genomically confirmed mother-neonate pairs.

**Methods:**

A single-center retrospective case-control study enrolled 43 mother-neonate pairs with genome sequencing-confirmed clonal *E. coli* vertical transmission (average nucleotide identity, ANI ≥99.99%) between January 2020 and June 2025. Pairs were stratified into an invasive infection group (n = 27) and a colonization group (n = 16) based on neonatal clinical outcomes. Genomic characterization, including multilocus sequence typing (MLST), serotyping, and virulence gene profiling, was performed on maternal *E. coli* isolates. The Boruta algorithm was used for feature selection, and a Bayesian logistic regression model was constructed to identify independent predictors of invasive infection.

**Results:**

No significant differences were observed in baseline clinical characteristics between the two groups. ST95 was exclusively detected in the invasive infection group (29.6% vs. 0%, *P* = 0.018). The virulence genes *neuA* (63% vs. 13%), *neuS* (59% vs. 13%), *iutA* (78% vs. 38%), and *kpsMT* II (93% vs. 63%) were significantly more prevalent in the invasive infection group (all raw *P* < 0.05). Bayesian modeling identified four independent predictors: ST95 [odds ratio (OR) = 6.05, 90% credible interval (CrI): 0.82–66.7, posterior probability P(OR>1) = 0.85], *neuA* (OR = 4.95, 90%CrI: 1.65–16.44, *P*(OR>1) = 0.96), *kpsMT* II (OR = 4.48, 90%CrI: 1.35–16.44, *P*(OR>1) = 0.95), and *iutA* (OR = 3.32, 90%CrI: 1.22–9.03, *P*(OR>1) = 0.93). Notably, all ST95 isolates (100%) co-harbored *neuA*, *kpsMT* II, and *iutA*, forming a distinct “clonal virulence complex.” The model exhibited good predictive performance (AUC = 0.847, 95%CI: 0.727–0.954), while the composite virulence burden score showed no intergroup difference (*P* = 0.208).

**Conclusion:**

The ST95 *E. coli* clonal virulence complex (co-harboring *neuA*/kpsMT II and *iutA*) is strongly associated with invasive infection following vertical transmission. This signature represents a potential candidate marker for clinical risk stratification of neonates at high risk of *E. coli* invasive infection, with potential implications for precision monitoring and intervention in perinatal care.

## 1 Introduction

Invasive Gram-negative bacterial infections are the leading cause of neonatal mortality and long-term neurodevelopmental sequelae worldwide (GBD MACO, 2016; Wen et al., 2024), with *Escherichia coli* (*E. coli*) being the most common pathogenic agent, accounting for approximately 34.6% of neonatal Gram-negative invasive infections (Hallmaier-Wacker et al., 2023). As a major pathogen of early-onset neonatal sepsis and severe pneumonia, *E. coli* has a high incidence and mortality in preterm and low birth weight infants, which poses a huge challenge to perinatal care (Lai et al., 2021; Mendoza-Palomar et al., 2017; Schrag et al., 2016; Sikias et al., 2023; Stoll et al., 2020). Vertical mother-to-neonate transmission is the critical route of early-onset *E. coli* infection, which can occur through placental hematogenous transmission, ascending infection of ruptured membranes, or birth canal exposure (Hwang et al., 2024; Klassert et al., 2020).

A fundamental unresolved clinical question is that only a subset of neonates develop life-threatening invasive infection after confirmed *E. coli* vertical transmission, while most neonates only present with asymptomatic local colonization (Li et al., 2025). Host immune status, obstetric factors, and antimicrobial exposure are considered to affect the infection outcome (Ullah et al., 2023), but the intrinsic bacterial molecular features that distinguish invasive infection from asymptomatic colonization remain poorly understood. From the pathogen-centric perspective, the pathogenic potential of *E. coli* is governed by its virulence factors and genetic background. Previous studies have identified various virulence genes (e.g., K1 capsule genes, iron acquisition genes) and high-risk sequence types (STs) associated with invasive *E. coli* infections (Daga et al., 2019; Gu et al., 2022), but most studies compare invasive strains with commensal or environmental strains, and lack systematic comparisons of maternal strains leading to different clinical outcomes (invasion vs colonization) in mother-neonate pairs with confirmed vertical transmission.

In addition, there is an ongoing debate regarding whether the overall quantity of virulence factors or specific virulence components predominantly determines *E. coli* pathogenicity. Some studies support a positive correlation between cumulative virulence gene count and invasive capacity, whereas others emphasize the critical role of synergistic interactions among distinct virulence modules (Li et al., 2023; Messika et al., 2012; Rasko et al., 2008). However, this controversy has not been investigated specifically among neonates with molecularly confirmed *E. coli* vertical transmission, resulting in a lack of reliable molecular evidence to support clinical risk stratification in this population.

To address these gaps, we conducted a retrospective case–control study focusing on mother–neonate pairs with genome sequencing-confirmed clonal *E. coli* vertical transmission. The study aimed to: (1) identify bacterial molecular features associated with divergent neonatal outcomes (invasive infection versus asymptomatic colonization) following vertical transmission; and (2) explore whether bacterial genetic backgrounds and virulence-related characteristics correlate with disease severity in this homogeneous transmission cohort. We hypothesized that clinical outcomes after perinatal *E. coli* vertical transmission may be associated with bacterial genetic lineages and their virulence profiles, while the relative contribution of cumulative virulence burden versus distinct virulence combinations remains to be distinguished within this clinical setting.

## 2 Materials and methods

### 2.1 Study design and participants

A single-center retrospective case-control study was conducted at the Women and Children’s Hospital, School of Medicine, Xiamen University. The study population included mother-neonate pairs with *E. coli* vertical transmission from January 2020 to June 2025. The source cohort consisted of 66,119 delivering women and 68,975 neonates hospitalized in our hospital during the same period. Placental swab cultures were collected from mothers with chorioamnionitis, urinary tract infection or other infectious diseases within 72 hours before delivery. Neonates were monitored according to the Expert Consensus on Diagnosis and Management of Neonatal Bacterial Sepsis (2024) (Subspecialty Group Of Neonatology TSOP and Editorial Board CJOP, 2024), and gastric fluid, blood and endotracheal secretion cultures were collected as required. The study complied with ethical requirements and strictly adhered to the principles of the Declaration of Helsinki and was approved by the Ethics Committee of the Women and Children’s Hospital affiliated with Xiamen University (KY-2023-063-H02). The use of de-identified retrospective data was granted an exemption from informed consent by the Ethics Committee.

#### 2.1.1 Inclusion criteria

**Figure 1:**
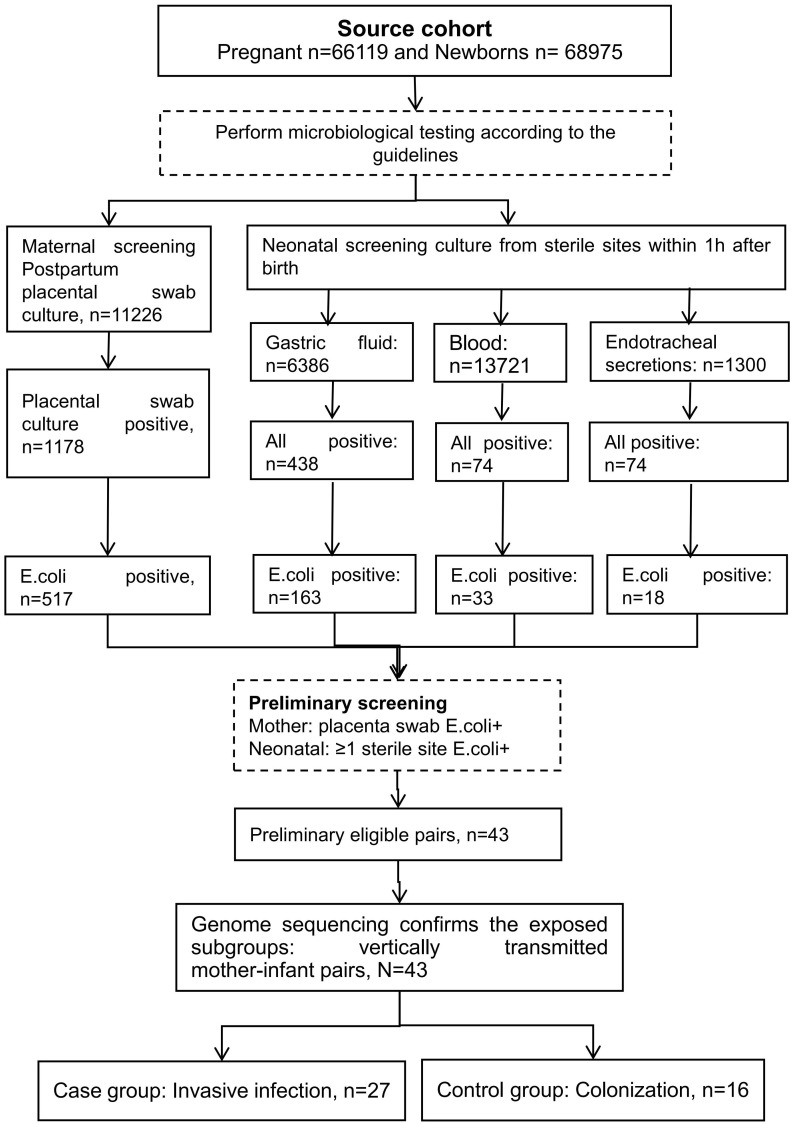
Study flow diagram. Flowchart illustrating the screening process from the source cohort (66,119 pregnant women and 68,975 neonates) to the final inclusion of 43 molecularly confirmed mother-neonate pairs with *E. coli* vertical transmission, and their classification into the invasive infection group (n=27) and colonization group (n=16). The diagram details the number of specimens collected, culture-positive rates, and exclusion criteria at each screening stage.

All of the following conditions must be met: ① Mothers with *E. coli*-positive placental swab culture; ② Neonates with at least one *E. coli*-positive sterile site culture (gastric fluid, blood or endotracheal secretion) within 1 hour after birth; ③ Mother-neonate pairs with clonal *E. coli* transmission confirmed by whole-genome sequencing, defined as ≤10 core genome single nucleotide polymorphisms (SNPs) between maternal and neonatal isolates, and additionally confirmed by an average nucleotide identity (ANI) ≥99.99% (FastANI v1.33, --fragLen 1000); ④ Complete clinical data of mothers and neonates available. The detailed screening process, including the number of specimens collected, culture-positive rates, and exclusion criteria at each stage, is illustrated in **Figure 1**.

#### 2.1.2 Exclusion criteria

① Neonates with enteral nutrition initiated before gastric fluid collection or endotracheal medication administered before endotracheal secretion collection; ② Mother-neonate pairs with non-clonal *E. coli* transmission or clinical data missing ≥30%; ③ Neonates complicated with congenital malformations, immunodeficiency.

#### 2.1.3 Group

A total of 517 maternal placental swab isolates and 182 neonatal isolates (164 gastric fluid + 18 tracheal secretion) were initially identified as *E. coli*. Among these, whole-genome sequencing was performed on all isolates from 89 mothers and neonates pairs who met the clinical sampling criteria for potential vertical transmission. After ANI and core-genome SNP analysis, 43 mother-neonate pairs met the clonal transmission criteria (≤10 core SNPs, ANI ≥99.99%) and were included in the final analysis. The remaining 46 pairs did not meet the clonal threshold and were excluded. According to the clinical outcomes, the enrolled pairs were divided into two groups: invasive infection group [infants with *E. coli*-positive blood/endotracheal secretion culture and clinical manifestations of invasive infection (Subspecialty Group Of Neonatology TSOP and Editorial Board CJOP, 2024; XM et al., 2017)] and colonization group (infants with only *E. coli*-positive gastric fluid culture, negative blood/endotracheal secretion culture, and no infection-related clinical symptoms with 72-hour follow-up without progression). A total of 43 mother-neonate pairs meeting the criteria were included in the final analysis (27 in the invasive infection group and 16 in the colonization group), with all samples included due to the rarity of genome-confirmed vertical transmission cases.

#### 2.1.4 Sample size

All 43 molecularly confirmed mother-neonate pairs were included in the final analysis, with the colonization group serving as the control for comparison against the invasive infection group.

### 2.2 Specimen processing and laboratory analysis

#### 2.2.1 Sample collection and bacterial isolation

Clinical specimens (maternal placental swabs; neonatal gastric fluid, blood, or tracheal secretions) meeting the study criteria were collected between January 2020 and June 30, 2025. Purified monoclonal *E. coli* isolates were preserved on filter paper and stored at –80 °C. Basic demographic and clinical information was collected.

#### 2.2.2 Bacterial identification and antimicrobial susceptibility testing

Before antimicrobial therapy, cultures of tracheal secretions and blood were performed on Columbia blood agar plates. Isolates were identified using the Phoenix™ 100 automated system. AST was performed using the disk diffusion method and interpreted according to the Clinical and Laboratory Standards Institute (CLSI) guidelines. *E. coli* ATCC 25922 was used as the quality control strain.

#### 2.2.3 Genomic DNA extraction

Genomic DNA from all isolates was extracted using the QIAamp^®^ DNA Mini Kit and stored at –20 °C.

#### 2.2.4 Library construction and genome sequencing

Genomic DNA was fragmented to approximately 400 bp, and sequencing libraries were prepared using the NEXTFLEX Rapid DNA-Seq Kit. Paired-end sequencing (2 × 150 bp) was performed on the Illumina NovaSeq™ X Plus platform.

#### 2.2.5 Genome assembly and annotation

The data generated from the Illumina platform were used for bioinformatics analysis. All analyses were performed using the Majorbio Cloud Platform (www.majorbio.com) from Shanghai Majorbio Bio-pharm Technology Co., Ltd. Raw reads were quality-filtered using fastp (v0.19.6) and assembled using SOAPdenovo2 (v2.04). Assembled genomes have been submitted to the NCBI database (accession numbers will be provided upon acceptance). Coding sequences (CDS) were predicted using Prodigal (v2.6.3). Functional annotation of predicted CDS was performed by alignment against the NR, Swiss-Prot, Pfam, GO, COG, KEGG, and CAZy databases using BLAST, Diamond, and HMMER (E-value < 1e-5). tRNAs and rRNAs were predicted using tRNAscan-SE (v2.0) and Barrnap (v0.9), respectively.

#### 2.2.6 *In silico* molecular characterization

Genome sequencing was performed on both maternal and neonatal *E. coli* isolates to confirm clonal transmission. After confirming clonal identity (ANI ≥99.99%, core genome SNPs ≤10), subsequent genomic characterization (MLST, serotyping, virulence gene profiling, etc.) was performed using the maternal isolates as the representative strain for each mother-neonate pair.

##### 2.2.6.1 Serotyping and phylogenetic grouping

Serotypes (O and H antigens) were determined using SerotypeFinder (v2.0) (https://cge.food.dtu.dk/services/SerotypeFinder/) with default parameters on the assembled genome sequences of all isolates. ClermonTyping software was used for phylogenetic group assignment (A, B1, B2, C, D, E, F, etc.).

##### 2.2.6.2 Multilocus sequence typing

MLST was performed by extracting sequences of seven housekeeping genes (*adk*, *fumC*, *gyrB*, *icd*, *mdh*, *purA*, *recA*) and comparing alleles against the PubMLST database to determine the sequence type (ST).

##### 2.2.6.3 Core-genome MLST

cgMLST analysis was performed using the EnteroBase *E. coli* cgMLST scheme via the cgMLST Finder tool.

##### 2.2.6.4 Virulence gene detection

Predicted CDS were aligned against the Virulence Factor Database (VFDB, v20240301) using Diamond (E-value ≤ 1e-5).

#### 2.2.7 Composite virulence burden score calculation

The composite virulence burden score was calculated by two dimensions with operon redundancy correction to avoid over counting (Johnson et al., 2015; van Hout et al., 2020): ① Gene abundance (S1) = number of positive virulence genes (one operon counted as one unit)/30, the denominator 30 represents the total number of unique virulence genes (after operon redundancy correction) that were screened in this study, based on the VFDB set of *E. coli* virulence genes. ② Functional diversity (S2) = number of positive functional categories (adhesion, toxins, iron acquisition, capsule/serum resistance, invasion/other)/5, the ‘invasion/other’ category included the following genes: *ibeA*, *ompA*, *tsh*, and *TraJ*. ③ CVB score = S1 + S2 (range 0-2).

### 2.3 Data collection

Clinical data of mothers and neonates were retrospectively extracted from the electronic medical record system, including maternal age, gestational age, delivery mode, premature rupture of membranes (PROM>18h), prenatal systemic antibiotic use within 72 hours, underlying diseases; neonatal sex, birth weight, gestational age stratification, Apgar score, clinical symptoms, laboratory test results, infection diagnosis and follow-up outcomes. Data extraction was performed by two independent researchers. Missing data with a missing rate of less than 5% were processed by complete case analysis.

### 2.4 Statistical analysis

All statistical analyses were performed using R v4.3.2 (packages: brms, bayestestR, Boruta, ggplot2). Continuous variables were expressed as median (interquartile range) [M(IQR)] and compared by Mann-Whitney U test; categorical variables were expressed as n(%) and compared by Chi-squared test or Fisher’s exact test. Statistical significance was defined as two-sided *P* < 0.05. For multiple comparisons, the Benjamini-Hochberg false discovery rate (FDR) correction was applied, with FDR < 0.05 considered significant.

Univariate comparisons with FDR correction were used for initial variable screening (*P* < 0.1 before correction). Variables with variance inflation factor (VIF) >10 were examined for collinearity. The Boruta algorithm, which is robust to multiple testing by using random shadow features, was then applied for final feature selection. The selected features were entered into a Bayesian logistic regression model, where inference is based on posterior probabilities and credible intervals rather than frequentist p−values, reducing concerns about multiple testing over fitting. Bayesian logistic regression model was applied to identify independent predictors of neonatal invasive infection, with neonatal infection outcome as the dependent variable (0=colonization, 1=invasive infection) and Boruta-selected features as independent variables. Weakly informative normal priors were used for the model (regression coefficients: N(0,2.5), intercept: N(0,5)), with model convergence judged by Rhat<1.1 and effective sample size (ESS) ratio>0.5. The model output included posterior mean, standard deviation (SD), 10%/50%/90% credible interval (CrI), odds ratio (OR) and posterior probability P(OR>1). The analysis was largely exploratory, feature selection (Boruta) and model specification were guided by data−driven approaches. No pre−specified analysis plan was registered. Therefore, findings should be interpreted as hypothesis−generating and require independent validation.

Receiver operating characteristic (ROC) curve was used to evaluate the predictive efficacy of the model, with the area under the curve (AUC) and 95% confidence interval (CI) calculated. Three sensitivity analyses were performed to verify the robustness of the results: ① Prior sensitivity analysis (comparing N(0,1.0), N(0,4.0) and t-distribution priors); ② Interaction analysis (adding ST95*×iutA*, ST95*×neuA*, ST95*×kpsMT II* interaction terms, leave-one-out (LOO) cross-validation for model comparison); ③ Unmeasured confounding analysis (calculating E-value to assess the impact of unmeasured confounders).

## 3 Results

### 3.1 Cohort characteristics

During the study period, 66,119 delivering women were included, of whom 11,226 underwent placental swab culture. The overall culture positivity rate was 10.5% (1,178/11,226). Further bacterial identification revealed that *E. coli* accounted for 43.9% (517/1,178) of all positive specimens, representing a positivity rate of 4.6% (517/11,226) among all placental swabs tested.

A total of 68,975 neonates were born during the same period, with 13,721 admitted to the neonatal care unit. All admitted neonates underwent blood culture, yielding an *E. coli* positivity rate of 0.2% (33/13,721), among which the ESBL positivity rate was 30.3% (10/33). Concurrently, gastric aspirate cultures were obtained within 1 hour after birth from 6,386 neonates, showing an *E. coli* positivity rate of 2.6% (164/6,386). Additionally, tracheal secretion cultures were collected from 1,300 neonates, with an *E. coli* positivity rate of 1.4% (18/1,300); among these positive isolates, the ESBL positivity rate was 22.2% (4/18).

Among the 89 mother-neonate pairs with both maternal and neonatal *E. coli* isolates available for sequencing, 43 pairs (48.3%) met the predefined clonal transmission criteria (≤10 core SNPs, ANI ≥99.99%). The remaining 46 pairs showed greater genetic divergence and were excluded from further analysis. Notably, among the 16 colonization group pairs, all 16 (100%) met the clonal criteria, indicating that vertical transmission of an identical strain does not inevitably lead to invasive infection.

According to neonatal outcome, these pairs were divided into two groups: 27 pairs where neonates developed invasive infection constituted the invasive infection group, and 16 pairs where neonates had colonization only constituted the colonization group. No statistically significant differences were observed between the two groups in baseline characteristics, including sex, gestational age, birthweight, mode of delivery, premature rupture of membranes, and use of prenatal antibiotics (all *P* > 0.05; **Table 1**). Given the small sample size, formal statistical comparisons of baseline characteristics may be underpowered.

**Table 1:** Baseline clinical and demographic characteristics of mother-neonate pairs.

Characteristic	Overall, N = 43	Colonization, N = 16	Invasive infection, N = 27	P-value^1^
Gestational age (w), Median (IQR)	39.29 (35.64 – 40.43)	39.29 (36.00 – 40.11)	39.14 (34.79 – 40.54)	0.99
Birth weight (g), Median (IQR)	3,106 (2,389 – 3,452)	3,146 (2,463 – 3,435)	3,070 (2,189 – 3,477)	0.75
Sex, n (%)				0.78
Male	20 (47)	7 (44)	13 (48)	
Female	23 (53)	9 (56)	14 (52)	
Delivery, n (%)				0.91
Vaginal	21 (49)	8 (50)	13 (48)	
Caesarean section	22 (51)	8 (50)	14 (52)	
PROM>18h, n (%)	12 (28)	5 (31)	7 (26)	0.74
Antibiotic, n (%)	9 (21)	4 (25)	5 (19)	0.71

^1^ Pearson's Chi-squared test; Wilcoxon rank sum test; Fisher's exact test; PROM: premature rupture of membranes; Antibiotic: Maternal administration of systemic antibiotics within 72 hours prior to delivery. P−values are provided for descriptive purposes to illustrate baseline comparability between groups, they do not imply causal inference due to the observational design.

Among the 43 maternal isolates, serotypes H5 (28%, 12/43) and H7 (14%, 6/43) were most common, with phylogenetic group B2 predominating (70%, 30/43), the predominant STs were ST95 (19%, 8/43) and ST1193 (14%, 6/43), as shown in **Supplementary Figure 1**. cgMLST analysis was performed on maternal isolates (which represent each mother-neonate pair due to confirmed clonal identity, ANI = 100%, SNPs ≤10). Across the 43 pairs, 35 distinct cgMLST types were identified, with identical profiles within each pair, further supporting clonal transmission (**Supplementary Table 1**). The prevalent virulence genes were *papC* (100%), *ompA* (100%), *kpsFEDUCS* (100%), *iroN* (100%), *fyuA* (100%), and *fimH* (100%). The resistance rates in descending order were ampicillin (74%), tetracycline (56%), compound sulfamethoxazole (46%), and cefazolin (33%), as shown in **Supplementary Figure 2**.

To assess whether the maternal isolate faithfully represents the neonatal isolate for the studied virulence features, we compared the 30-gene virulence profiles between each mother-neonate pair. In 40 of 43 pairs (93.0%), the profiles were completely identical. In the remaining three pairs (all non-ST95), the maternal isolate carried one additional non-predictor virulence gene (*pic* or *sat*) not present in the neonatal isolate. Importantly, the four independent predictors (ST95, *neuA*, *kpsMT II*, *iutA*) were 100% concordant in all pairs. These data confirm that the maternal isolates reliably represent the neonatal isolates for the key findings of this study.

### 3.2 Univariate analysis of bacterial molecular features

Univariate analysis revealed significant differences in the prevalence of several virulence genes and STs between groups (**Supplementary Table 1**). ST95 prevalence was significantly higher in the infection group [29.6% (8/27)] than in the colonization group [0% (0/16); raw *P* = .0.018, FDR-corrected *P* = 0.036]. The positivity rates for *neuA* (63% vs 13%, *P* = 0.001, FDR-corrected *P* = 0.010), *neuS*(59% vs 13%, *P* = 0.003, FDR-corrected *P* = 0.011), *iutA* (78% vs 38%, *P* = 0.008, FDR-corrected *P* = 0.022), and *kpsMT II* (93% vs 63%, *P* = 0.037, FDR-corrected *P* = 0.059)were significantly higher in the infection group (all raw *P* < 0.05). Among eight variables with P<0.1 (*neuA*, *neuS*, *iutA*, *kpsMT II*, H7, ST95, *chuA*, *papA*, as shown in **Supplementary Table 5**), variance inflation factor analysis indicated severe multicollinearity between *neuA* (VIF 17.68) and neuS (VIF 18.37). Consequently, *neuS* was excluded, and the remaining variables were subjected to Boruta feature selection (**Supplementary Figure 3**).

### 3.3 Association of ST95 with key virulence genes

**Figure 2:**
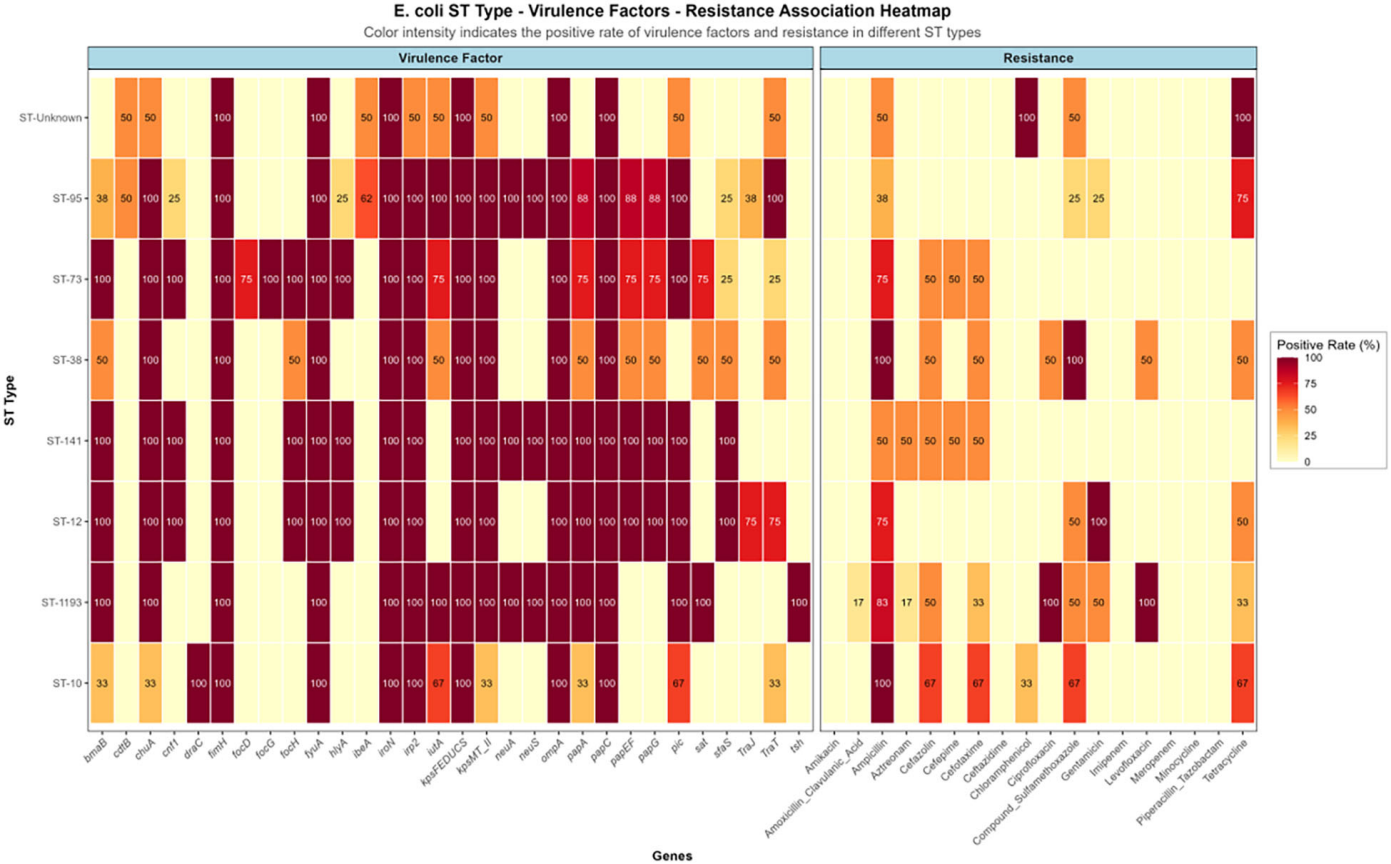
Heatmap of key virulence gene presence across ST95 and non-ST95 isolates. Heatmap showing the presence (dark blue) or absence (light blue) of selected virulence genes (*neuA, kpsMT II*, *iutA*, etc.) in ST95 isolates (left cluster, n=8) versus non-ST95 isolates (right cluster, n=35). The dashed box highlights the 100% co-carriage of *kpsMT II* and *iutA* in all ST95 isolates, with *neuA* also uniformly present in this clonal group.

**Figure 3:**
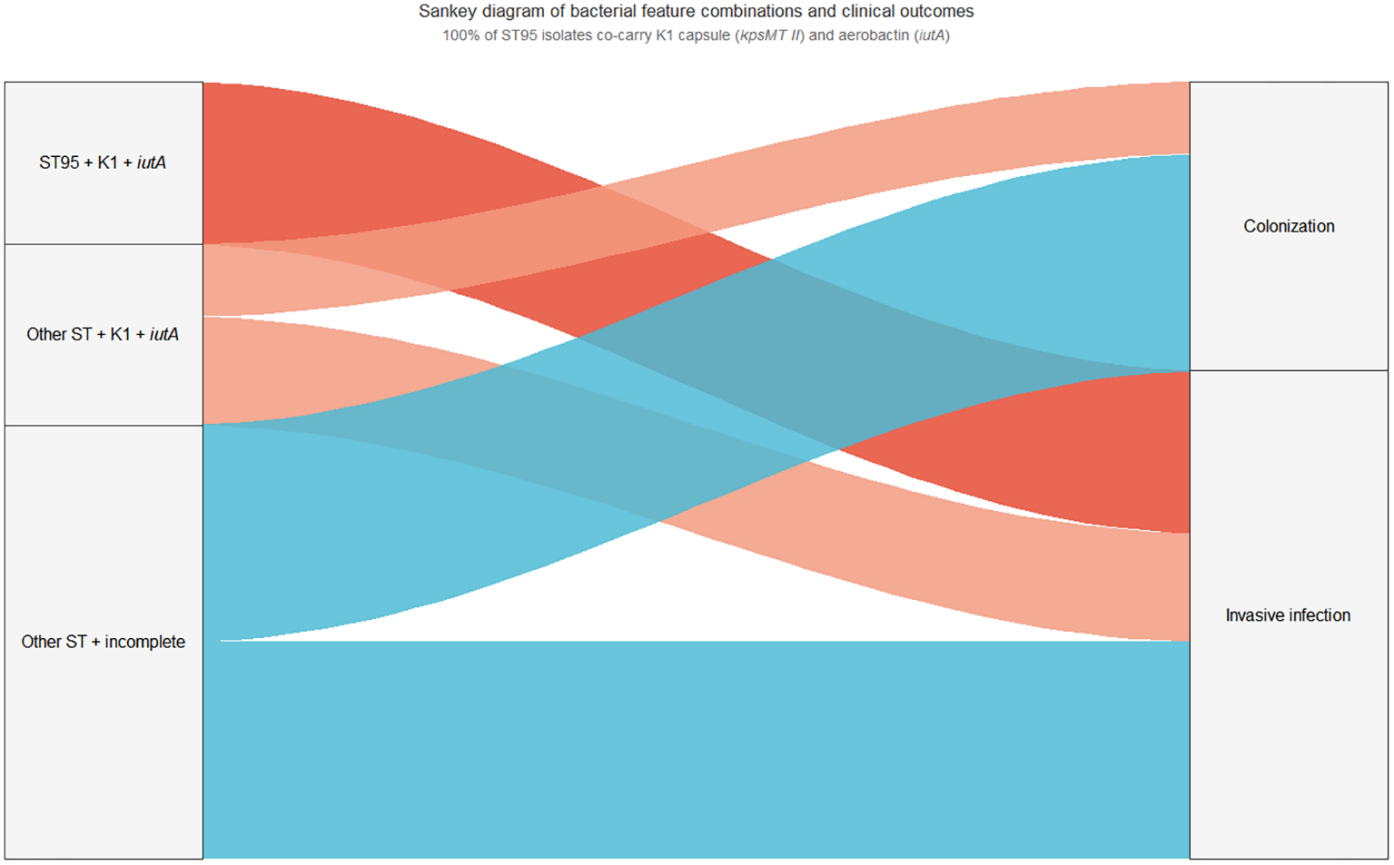
Sankey diagram illustrating the flow from bacterial characteristics to clinical outcome. Sankey diagram depicting the associations between Sequence Types (ST95, ST1193, Others), the carriage of key virulence modules (K1 capsule: *neuA*/*kpsMT II*, Aerobactin: *iutA*), and the final clinical outcome (Invasive Infection, Colonization). The width of each link is proportional to the number of isolates, with thicker links indicating larger sample sizes. Given the small sample size, the widths are intended for visual pattern illustration rather than statistical inference. ‘Incomplete’ refers to isolates of sequence types other than ST95 that do not carry all three key virulence genes (*neuA*, *kpsMT II*, and *iutA*) simultaneously.

Analysis of co-occurrence revealed that all ST95 isolates (8/8, 100%) simultaneously carried the Group 2 capsule marker (*kpsMT II*), the aerobactin gene (*iutA*), and the K1/K92-specific sialic acid synthesis gene (*neuA*). Among non-ST95 isolates (n = 35), the positivity rates for *kpsMT II*, *iutA*, and *neuA* were 77.1% (27/35), 54.3% (19/35), and 31.4% (11/35), respectively (**Figure 2**). This indicates that ST95 characterizes a high-risk clonal complex stably co-expressing the K1 capsule and a high-efficiency iron acquisition system in this cohort. The ST95 background, through its carriage of specific virulence genes, showed a strong association with invasive outcome, whereas individual virulence genes lacked absolute specificity (**Figure 3**).

### 3.4 Bayesian logistic regression identifying independent predictors

The Bayesian logistic regression model identified four independent bacterial predictors (**Table 2**). ST95 showed the strongest association with invasive outcome [posterior median odds ratio (OR) 6.05, 90% credible interval (CrI) 0.82–66.7; with a posterior probability of 85% that the true OR exceeds 1 (P(OR>1)=0.85)]. This indicates substantial, though not conclusive, Bayesian evidence supporting a positive association between ST95 carriage and invasive infection. Similar probabilistic interpretations apply to other predictors: *neuA* [OR 4.95, 90% CrI 1.65–16.44; P(OR>1)=0.96], *kpsMT II* [OR 4.48, 90% CrI 1.35–16.44; P(OR>1)=0.95], and *iutA* [OR 3.32, 90% CrI 1.22–9.03; P(OR>1)=0.93], all exhibiting very high posterior probabilities (>0.93) favoring a positive effect. The model demonstrated good discrimination (AUC 0.847, 95% CI 0.727–0.954) (**Supplementary Figure 4**). Posterior predictive checks showed the mean predicted infection probability (0.60) closely matched the observed rate (62.8%). Notably, all ST95 isolates (8/8) co-carried *kpsMT II* and *iutA*. In the invasive infection group, 9 isolates (33.3%) simultaneously carried *kpsMT II* and *iutA*; among these, 8 were ST95 isolates (representing 100% of all ST95 isolates in this cohort), and one non-ST95 isolate also carried both genes. Notably, all 8 ST95 isolates additionally carried *neuA*, forming a complete ST95-K1-aerobactin complex. No isolate in the colonization group possessed all three features *(kpsMT II*, *iutA*, and ST95) (*P* < 0.001).

**Table 2:** Independent bacterial molecular predictors of neonatal invasive E. coli infection identified by Bayesian logistic regression.

Characteristic	Mean	SD	Crl			Rhat	ESS ratio	MCSE	OR	P(OR>1)*
			10%	50%	90%					
Intercept	-2.1	1.0	-3.4	-2.0	-0.8	1.0	0.83	0.0	0.12	0.02
neuA	1.7	0.9	0.5	1.6	2.8	1.0	0.78	0.0	4.95	0.96
ST95	1.9	1.8	-0.2	1.8	4.2	1.0	0.61	0.0	6.05	0.85
iutA	1.2	0.8	0.2	1.2	2.2	1.0	0.90	0.0	3.32	0.93
kpsMT II	1.5	1.0	0.3	1.5	2.8	1.0	0.84	0.0	4.48	0.95

*P (OR>1) indicates a posterior probability of odds ratio greater than 1. Crl: Credible interval. sd: Standard Deviation. Rhat: R-hat (Gelman-Rubin). ESS ratio: Effective Sample Size Ratio. mcse: Monte Carlo Standard Error. OR: Odds Ratio.

### 3.5 Sensitivity analysis

#### 3.5.1 Comparison of different prior distributions

Sensitivity analysis (**Supplementary Table 2**) showed that the effect directions of key predictors (ST95, *kpsMT II*, *neuA*, *iutA*) remained consistent even when using more conservative (N (0,1.0)) or more diffuse (N (0,4.0)) priors, or when using t-distribution priors, indicating that the main conclusions were not overly sensitive to prior specification.

#### 3.5.2 Exploratory interaction analysis

Interaction terms (ST95 × *iutA*, ST95 × *neuA* and ST95 × *kpsMT II*) were added to explore potential synergy. The coefficient estimates for all interaction terms were positive (median log-OR = 0.60), yet their 95% credible intervals contained zero, indicating they did not reach conventional statistical significance. Leave-one-out cross-validation comparison showed that models containing any of these interaction terms (elpd diff = 0.0) performed similarly to, or very marginally better than, the baseline model without interactions (elpd diff = -0.1); however, this difference was smaller than its standard error (SE = 0.1), as shown in **Supplementary Table 3**.

#### 3.5.3 Assessment of unmeasured confounding

E-values calculated based on the posterior distribution are shown in **Supplementary Table 4**. Despite wide confidence intervals due to sample size, the E-values for point estimates were high (*neuA*: 9.73, ST95: 11.19, *iutA*: 6.17, *kpsMT II*: 8.4). This suggests that an unmeasured confounder would need to be strongly associated with both the exposure and the outcome to fully explain the observed associations, which is biologically challenging.

### 3.6 Exploratory analysis of CVB score

**Figure 4:**
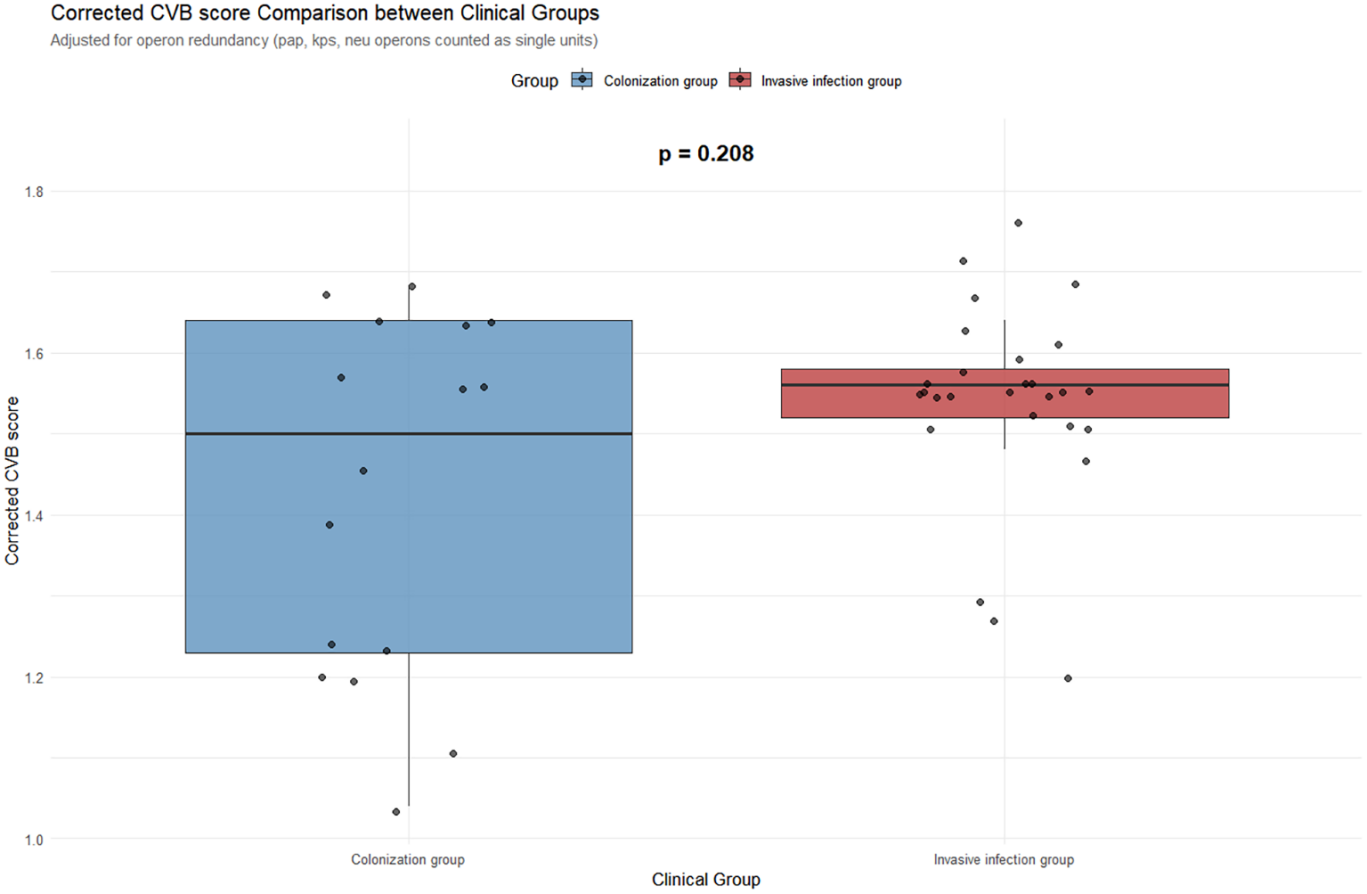
Boxplot comparing the composite virulence burden score (BHScore) between invasive infection and colonization groups. Boxplot showing the distribution of BHScore (range 0-2) in the invasive infection group (n=27) versus the colonization group (n=16). The box represents the interquartile range (IQR), the horizontal line within the box is the median, and the whiskers extend to the minimum and maximum values within 1.5×IQR. Mann-Whitney U test, P = 0.208; the adjusted P-value after operon redundancy correction is indicated.

Exploratory analysis was performed to assess whether the overall virulence gene burden was associated with invasive infection. The CVB score showed no significant difference between the invasive infection group and the colonization group (W = 166.5, *P* = 0.208; **Figure 4**). Wide within-group variability was observed in both groups.

## 4 Discussion

This retrospective case-control study, combined with Bayesian modeling, investigated bacterial molecular determinants associated with invasive *E. coli* infection among 43 mother–neonate pairs with genomically confirmed clonal vertical transmission. The composite virulence burden score showed no discriminatory value between groups (P = 0.208). In contrast, the Bayesian model incorporating ST95, *neuA*, *kpsMT II*, and *iutA* achieved good predictive performance (AUC = 0.847). Together, these findings suggest that neonatal infection outcome is determined by the synergy between high-risk clonal background and specific virulence modules, rather than by the cumulative number of virulence genes. Given the exploratory nature of the analysis, these results should be considered hypothesis-generating and warrant further validation in independent cohorts.

The ST95-K1-aerobactin clonal virulence complex is the key mediator of invasive infection following vertical transmission, with its pathogenic synergy robustly supported by numerous *in vitro* and *in vivo* functional validation studies (Nhu et al., 2024; Riley, 2014; Xia et al., 2022; Xiao et al., 2023). ST95 was not detected in the colonization group in this cohort (0/16), which is consistent with its previously described enrichment in invasive disease. As a globally recognized high-risk lineage associated with neonatal meningitis and sepsis, this observation underscores its unique invasive potential, endowing *E. coli* with enhanced host immune evasion and systemic dissemination capacity beyond local colonization, a trait experimentally validated by studies demonstrating ST95 clones’ superior endothelial cell invasion and blood-brain barrier penetration (Nhu et al., 2024). However, given the small sample size of the colonization group, we cannot exclude the possibility that ST95 may occasionally be found in asymptomatic colonized neonates, as suggested by larger surveillance studies that have detected ST95, albeit at low prevalence (Gladstone et al., 2026). The core mechanistic synergy of this complex stems from the coordinated action of the K1 capsule and aerobactin system, with the ST95 clonal background unifying these two virulence modules into a triple pathogenic synergy of “immune escape-iron uptake-intrinsic invasiveness” via stable 100% co-carriage. The K1 capsule is encoded by a dedicated capsule biosynthesis gene cluster (including *kpsFEDUCS*, *neuDBACES*, and *kpsMT*), for which *neuA* and *kpsMT II* serve as useful genetic markers. The K1 capsule mediates immune evasion by resisting phagocytosis, inhibiting complement C3 deposition, and evading macrophage attack (Gao et al., 2024); its well-documented neurotropism forms the critical basis for bacterial survival in the neonatal bloodstream. Complementarily, the aerobactin iron acquisition system encoded by *iutA* exhibits the highest iron uptake efficiency among all characterized *E. coli* siderophore systems and acts as a pivotal survival factor in the iron-limited neonatal host microenvironment (Sharp and Foster, 2025). This stable co-carriage forms a self-reinforcing “pathogenic synergy loop”: K1-mediated immune escape enables persistent bloodstream survival, which in turn allows the aerobactin system to exploit host iron limitation to support bacterial proliferation, ultimately driving systemic invasive infection rather than local colonization.

Our study further does not support the traditional virulence gene-count paradigm: the non-discriminatory composite virulence burden score (CVBscore, P = 0.208) suggests that virulence gene quantity is irrelevant to pathogenic potential in a strictly clonal transmission cohort. The wide within-group variability observed in **Figure 4** indicates that cumulative virulence gene burden varies considerably among individual isolates within the same clinical outcome group. This overlapping distribution further supports that a simple count of virulence genes does not capture pathogenic potential, as some colonizing isolates may carry many virulence genes yet not cause disease, while some invasive isolates may carry fewer but functionally potent combinations. Given the modest sample size, we cannot perform robust subgroup analyses to explore the sources of variability, nor can we rule out a small effect. Larger studies are needed to confirm the absence of association and to assess whether the CVB score might have discriminatory power in specific subgroups. Instead, invasive capacity is dictated by specific synergistic virulence modules within a high-risk clonal background (ST95). This finding highlights the critical regulatory role of bacterial genetic background in modulating virulence gene function, refining the classical understanding of *E. coli* pathogenicity and establishing a unified “Clone-Virulence Module” framework for interpreting neonatal *E. coli* infection (Leimbach et al., 2013; Vanstokstraeten et al., 2022). Recent large-scale genomic evidence further demonstrates that stable associations between chromosomal clonal lineages and plasmid-encoded virulence or competitive traits are central to clonal success and pathogenic potential in extraintestinal pathogenic *E. coli* (Arredondo-Alonso et al., 2025). In essence, our results provide clinical cohort validation of these well-characterized molecular mechanisms within the unique setting of *E. coli* vertical transmission, and suggest that the ST95-K1-aerobactin clonal virulence complex acts as a clinically relevant driver of neonatal invasive infection.

The ST95-K1-aerobactin complex represents a potential candidate marker. The absence of Group 1 and Group 4 capsule loci in our cohort contrasts with findings from Gladstone et al. in low- and middle-income countries (Gladstone et al., 2026), possibly reflecting regional differences in circulating lineages, sampling strategies (placental swabs from symptomatic mothers vs. general colonization), or the vertical transmission context. Therefore, our signature requires validation in geographically diverse populations before generalization. From a clinical translational perspective, the combination of *neuA* and *iutA* (both detectable by targeted PCR) is more feasible than full ST95 typing (MLST/WGS). In our cohort, all ST95 isolates were *neuA*+/*iutA*+, and no ST95 isolate lacked either gene. Adding *kpsMT II* offers little discriminatory value, as Group 2 capsules are common in carriage (Gladstone et al., 2026). Thus, a *neu*A+/*iutA*+ dual-positive PCR result could serve as a practical risk stratification tool for neonates following vertical transmission, capturing the functionally relevant components of the ST95-K1-aerobactin complex without requiring ST95 typing. This hypothesis requires prospective validation and could inform future studies on enhanced monitoring or tailored antibiotic strategies, but empirical use of broad-spectrum agents such as carbapenems is not currently recommended. Integration into prenatal screening protocols could be considered in future research to assess its utility in reducing vertical transmission and neonatal morbidity.

Several limitations must be acknowledged. ① Single-center, retrospective design: The small sample size (43 pairs), imposed by the strict inclusion criterion of clonal transmission, leads to wide credible intervals for ST95. Consequently, the absence of statistically significant differences in baseline clinical characteristics (all *P* > 0.05) should not be interpreted as evidence of equivalence. Multicenter prospective validation is required to confirm the generalizability of our risk signature. ② Bacterial-centric focus: The study does not incorporate host factors, which are critical determinants of infection outcome. Future studies must integrate host-microbiome interactions to build more comprehensive models. ③ Lack of functional validation: *In vitro*/*in vivo* experiments are needed to directly confirm the invasive advantage conferred by the ST95-K1-aerobactin complex. ④The composite virulence burden score assigned equal weight to all functional categories, which may not reflect biological importance; future studies could incorporate weighted scoring based on functional impact. The fact that all colonization pairs also exhibited clonal transmission underscores that strain identity alone is insufficient to determine clinical outcome, reinforcing the importance of specific virulence modules. ⑤It should be noted that maternal *E. coli* isolates were obtained from women with clinical signs of infection; Therefore, the strains we compared already possessed virulence traits. Our study design does not address whether a true commensal strain can acquire such traits and transition to invasive infection – that question requires a different experimental approach. ⑥Clinical feasibility assessment: The practicality of our molecular signature for routine prenatal/neonatal screening requires further evaluation. ⑦Our study design does not allow estimation of the negative predictive value of the ST95−K1−aerobactin signature, the proportion of exposed infants who remain uninfected or even uncolonized. Future prospective cohort studies that screen all mothers with *E. coli*−positive placental swabs and follow all neonates regardless of infection status are needed to assess the clinical utility of this signature for risk stratification.

## 5 Conclusion

In mother–neonate pairs with genomically confirmed clonal vertical transmission of *E. coli*, a high-risk clonal background ST95 carrying the K1 capsule and aerobactin iron acquisition system is strongly associated with neonatal invasive infection. Our findings refute the traditional virulence gene-count paradigm and support a Clone-Virulence Module framework, in which specific virulence determinants within a high-risk lineage determine invasive potential rather than cumulative virulence burden. The ST95-K1-aerobactin signature represents a potential candidate marker that warrants further prospective multicenter validation and mechanistic studies to translate it into clinical practice.

## Data Availability

The datasets presented in this study can be found in online repositories. The names of the repository/repositories and accession number(s) can be found below: https://www.ncbi.nlm.nih.gov/, PRJNA1395677/SRP659051.
