# Supplementary material for: Molecular determinants of Escherichia coli causing neonatal invasive infection following vertical transmission
Source: Front Cell Infect Microbiol. 2026 Jun 15;16:1855839. doi: 10.3389/fcimb.2026.1855839 (PMC13310911; doi:10.3389/fcimb.2026.1855839)
Supplement: Supplementary file 5 [file Table1.docx]

**Supplementary Table 1: Detailed comparison of all molecular and phenotypic features between invasive infection and colonization groups**

| **Characteristic** | **Overall**, N = 43 | Colonization, N = 16 | Invasive infection, N = 27 | **p-value** |
| --- | --- | --- | --- | --- |
| **Serotype (O antigens)** |  |  |  |  |
| Gp2(O28ac/O42), n (%) | 1 (2.3) | 1 (6.3) | 0 (0) | 0.37 |
| Gp7（O50,O2）, n (%) | 5 (12) | 1 (6.3) | 4 (15) | 0.64 |
| O1, n (%) | 1 (2.3) | 0 (0) | 1 (3.7) | >0.99 |
| O131, n (%) | 1 (2.3) | 0 (0) | 1 (3.7) | >0.99 |
| O153, n (%) | 1 (2.3) | 0 (0) | 1 (3.7) | >0.99 |
| O16, n (%) | 1 (2.3) | 0 (0) | 1 (3.7) | >0.99 |
| O18, n (%) | 6 (14) | 1 (6.3) | 5 (19) | 0.39 |
| O25, n (%) | 3 (7.0) | 0 (0) | 3 (11) | 0.28 |
| O4, n (%) | 4 (9.3) | 3 (19) | 1 (3.7) | 0.14 |
| O45, n (%) | 1 (2.3) | 1 (6.3) | 0 (0) | 0.37 |
| O5, n (%) | 1 (2.3) | 0 (0) | 1 (3.7) | >0.99 |
| O6, n (%) | 7 (16) | 4 (25) | 3 (11) | 0.39 |
| O7, n (%) | 1 (2.3) | 0 (0) | 1 (3.7) | >0.99 |
| O75, n (%) | 3 (7.0) | 0 (0) | 3 (11) | 0.28 |
| O78, n (%) | 1 (2.3) | 1 (6.3) | 0 (0) | 0.37 |
| O83, n (%) | 1 (2.3) | 0 (0) | 1 (3.7) | >0.99 |
| O86, n (%) | 1 (2.3) | 1 (6.3) | 0 (0) | 0.37 |
| O9, n (%) | 1 (2.3) | 1 (6.3) | 0 (0) | 0.37 |
| Gp15(O101), n (%) | 1 (2.3) | 1 (6.3) | 0 (0) | 0.37 |
| ONT, n (%) | 2 (4.7) | 1 (6.3) | 1 (3.7) | >0.99 |
| **Serotype (H antigens)** |  |  |  |  |
| H1, n (%) | 4 (9.3) | 2 (13) | 2 (7.4) | 0.62 |
| H10, n (%) | 1 (2.3) | 1 (6.3) | 0 (0) | 0.37 |
| H15, n (%) | 1 (2.3) | 0 (0) | 1 (3.7) | >0.99 |
| H18, n (%) | 3 (7.0) | 2 (13) | 1 (3.7) | 0.54 |
| H2, n (%) | 1 (2.3) | 1 (6.3) | 0 (0) | 0.37 |
| H21, n (%) | 2 (4.7) | 1 (6.3) | 1 (3.7) | >0.99 |
| H28, n (%) | 1 (2.3) | 0 (0) | 1 (3.7) | >0.99 |
| H30, n (%) | 1 (2.3) | 1 (6.3) | 0 (0) | 0.37 |
| H31, n (%) | 2 (4.7) | 1 (6.3) | 1 (3.7) | >0.99 |
| H4, n (%) | 6 (14) | 1 (6.3) | 5 (19) | 0.39 |
| H5, n (%) | 12 (28) | 5 (31) | 7 (26) | 0.74 |
| H6, n (%) | 3 (7.0) | 1 (6.3) | 2 (7.4) | >0.99 |
| H7, n (%) | 6 (14) | 0 (0) | 6 (22) | 0.069 |
| **Phylogenetic group** |  |  |  |  |
| A, n (%) | 6 (14) | 4 (25) | 2 (7.4) | 0.17 |
| B1, n (%) | 3 (7.0) | 1 (6.3) | 2 (7.4) | >0.99 |
| B2, n (%) | 30 (70) | 9 (56) | 21 (78) | 0.18 |
| D, n (%) | 3 (7.0) | 2 (13) | 1 (3.7) | 0.54 |
| F, n (%) | 1 (2.3) | 0 (0) | 1 (3.7) | >0.99 |
| **Multilocus sequence typing** |  |  |  |  |
| ST10, n (%) | 3 (7.0) | 2 (13) | 1 (3.7) | 0.54 |
| ST101, n (%) | 1 (2.3) | 0 (0) | 1 (3.7) | >0.99 |
| ST1193, n (%) | 6 (14) | 1 (6.3) | 5 (19) | 0.39 |
| ST12, n (%) | 4 (9.3) | 3 (19) | 1 (3.7) | 0.14 |
| ST127, n (%) | 1 (2.3) | 1 (6.3) | 0 (0) | 0.37 |
| ST131, n (%) | 1 (2.3) | 0 (0) | 1 (3.7) | >0.99 |
| ST141, n (%) | 2 (4.7) | 0 (0) | 2 (7.4) | 0.52 |
| ST1485, n (%) | 1 (2.3) | 0 (0) | 1 (3.7) | >0.99 |
| ST156, n (%) | 1 (2.3) | 0 (0) | 1 (3.7) | >0.99 |
| ST38, n (%) | 2 (4.7) | 1 (6.3) | 1 (3.7) | >0.99 |
| ST43, n (%) | 1 (2.3) | 1 (6.3) | 0 (0) | 0.37 |
| ST4456, n (%) | 1 (2.3) | 0 (0) | 1 (3.7) | >0.99 |
| ST617, n (%) | 1 (2.3) | 1 (6.3) | 0 (0) | 0.37 |
| ST681, n (%) | 1 (2.3) | 1 (6.3) | 0 (0) | 0.37 |
| ST69, n (%) | 1 (2.3) | 1 (6.3) | 0 (0) | 0.37 |
| ST73, n (%) | 4 (9.3) | 2 (13) | 2 (7.4) | 0.62 |
| ST93, n (%) | 1 (2.3) | 0 (0) | 1 (3.7) | >0.99 |
| ST95, n (%) | 8 (19) | 0 (0) | 8 (30) | 0.018 |
| ST998, n (%) | 1 (2.3) | 1 (6.3) | 0 (0) | 0.37 |
| ST Unknown, n (%) | 2 (4.7) | 1 (6.3) | 1 (3.7) | >0.99 |
| **Core-genome MLST** |  |  |  |  |
| 114324, n (%) | 1 (2.3) | 1 (6.3) | 0 (0) | 0.37 |
| 116253, n (%) | 1 (2.3) | 0 (0) | 1 (3.7) | >0.99 |
| 117229, n (%) | 2 (4.7) | 2 (13) | 0 (0) | 0.13 |
| 117347, n (%) | 1 (2.3) | 0 (0) | 1 (3.7) | >0.99 |
| 119116, n (%) | 1 (2.3) | 0 (0) | 1 (3.7) | >0.99 |
| 119503, n (%) | 1 (2.3) | 0 (0) | 1 (3.7) | >0.99 |
| 119535, n (%) | 1 (2.3) | 0 (0) | 1 (3.7) | >0.99 |
| 119588, n (%) | 2 (4.7) | 1 (6.3) | 1 (3.7) | >0.99 |
| 133740, n (%) | 1 (2.3) | 1 (6.3) | 0 (0) | 0.37 |
| 147915, n (%) | 1 (2.3) | 1 (6.3) | 0 (0) | 0.37 |
| 147932, n (%) | 1 (2.3) | 1 (6.3) | 0 (0) | 0.37 |
| 159017, n (%) | 1 (2.3) | 0 (0) | 1 (3.7) | >0.99 |
| 161137, n (%) | 1 (2.3) | 0 (0) | 1 (3.7) | >0.99 |
| 1679, n (%) | 2 (4.7) | 0 (0) | 2 (7.4) | 0.52 |
| 176335, n (%) | 1 (2.3) | 0 (0) | 1 (3.7) | >0.99 |
| 182026, n (%) | 1 (2.3) | 0 (0) | 1 (3.7) | >0.99 |
| 187272, n (%) | 1 (2.3) | 1 (6.3) | 0 (0) | 0.37 |
| 188101, n (%) | 1 (2.3) | 1 (6.3) | 0 (0) | 0.37 |
| 192278, n (%) | 1 (2.3) | 0 (0) | 1 (3.7) | >0.99 |
| 198112, n (%) | 1 (2.3) | 1 (6.3) | 0 (0) | 0.37 |
| 206678, n (%) | 1 (2.3) | 1 (6.3) | 0 (0) | 0.37 |
| 29172, n (%) | 1 (2.3) | 1 (6.3) | 0 (0) | 0.37 |
| 32184, n (%) | 1 (2.3) | 0 (0) | 1 (3.7) | >0.99 |
| 40781, n (%) | 1 (2.3) | 0 (0) | 1 (3.7) | >0.99 |
| 4085, n (%) | 2 (4.7) | 0 (0) | 2 (7.4) | 0.52 |
| 43295, n (%) | 2 (4.7) | 0 (0) | 2 (7.4) | 0.52 |
| 52617, n (%) | 1 (2.3) | 0 (0) | 1 (3.7) | >0.99 |
| 62326, n (%) | 1 (2.3) | 0 (0) | 1 (3.7) | >0.99 |
| 70972, n (%) | 4 (9.3) | 3 (19) | 1 (3.7) | 0.14 |
| 75997, n (%) | 1 (2.3) | 0 (0) | 1 (3.7) | >0.99 |
| 80283, n (%) | 1 (2.3) | 0 (0) | 1 (3.7) | >0.99 |
| 8623, n (%) | 1 (2.3) | 1 (6.3) | 0 (0) | 0.37 |
| 88805, n (%) | 1 (2.3) | 0 (0) | 1 (3.7) | >0.99 |
| 92614, n (%) | 1 (2.3) | 0 (0) | 1 (3.7) | >0.99 |
| 96053, n (%) | 1 (2.3) | 0 (0) | 1 (3.7) | >0.99 |
| **Virulence gene** |  |  |  |  |
| Number of Virulence genes, Median (IQR) | 17.00 (14.00 – 20.00) | 14.50 (10.00 – 20.00) | 17.00 (15.00 – 19.50) | 0.11 |
| *fimH*, n (%) | 43 (100) | 16 (100) | 27 (100) |  |
| *papC*, n (%) | 43 (100) | 16 (100) | 27 (100) |  |
| *papA*, n (%) | 31 (72) | 9 (56) | 22 (81) | 0.092 |
| *papG*, n (%) | 21 (49) | 7 (44) | 14 (52) | 0.61 |
| *papEF*, n (%) | 20 (47) | 6 (38) | 14 (52) | 0.36 |
| *sfaS*, n (%) | 14 (33) | 6 (38) | 8 (30) | 0.59 |
| *focD*, n (%) | 4 (9.3) | 2 (13) | 2 (7.4) | 0.62 |
| *focG*, n (%) | 5 (12) | 2 (13) | 3 (11) | >0.99 |
| *focH*, n (%) | 14 (33) | 6 (38) | 8 (30) | 0.59 |
| *draC*, n (%) | 6 (14) | 4 (25) | 2 (7.4) | 0.17 |
| *bmaB*, n (%) | 29 (67) | 10 (63) | 19 (70) | 0.59 |
| *sat*, n (%) | 10 (23) | 3 (19) | 7 (26) | 0.72 |
| *ompA*, n (%) | 43 (100) | 16 (100) | 27 (100) |  |
| *tsh,* n (%) | 8 (19) | 1 (6.3) | 7 (26) | 0.22 |
| *chuA*, n (%) | 38 (88) | 12 (75) | 26 (96) | 0.056 |
| *iutA*, n (%) | 27 (63) | 6 (38) | 21 (78) | 0.008 |
| *fyuA*, n (%) | 43 (100) | 16 (100) | 27 (100) |  |
| *irp2,* n (%) | 41 (95) | 14 (88) | 27 (100) | 0.13 |
| *iroN*, n (%) | 43 (100) | 16 (100) | 27 (100) |  |
| *kpsFEDUCS*, n (%) | 43 (100) | 16 (100) | 27 (100) |  |
| *neuA*, n (%) | 19 (44) | 2 (13) | 17 (63) | 0.001 |
| *neuS*, n (%) | 18 (42) | 2 (13) | 16 (59) | 0.003 |
| *kpsMT II*, n (%) | 35 (81) | 10 (63) | 25 (93) | 0.037 |
| *TraT*, n (%) | 25 (58) | 7 (44) | 18 (67) | 0.14 |
| *cnf1*, n (%) | 14 (33) | 7 (44) | 7 (26) | 0.23 |
| *hlyA*, n (%) | 14 (33) | 7 (44) | 7 (26) | 0.23 |
| *cdtB*, n (%) | 5 (12) | 0 (0) | 5 (19) | 0.14 |
| *pic*, n (%) | 33 (77) | 10 (63) | 23 (85) | 0.14 |
| *ibeA*, n (%) | 8 (19) | 1 (6.3) | 7 (26) | 0.22 |
| *TraJ*, n (%) | 12 (28) | 5 (31) | 7 (26) | 0.74 |
| **Antimicrobial susceptibility testing** |  |  |  |  |
| ESBLs, n (%) | 11 (26) | 5 (31) | 6 (22) | 0.72 |
| Ampicillin, n (%) |  |  |  | 0.27 |
| R | 31 (74) | 13 (87) | 18 (67) |  |
| S | 11 (26) | 2 (13) | 9 (33) |  |
| Unknown | 1 | 1 | 0 |  |
| Cefazolin, n (%) |  |  |  | 0.92 |
| I | 11 (26) | 4 (25) | 7 (26) |  |
| R | 14 (33) | 6 (38) | 8 (30) |  |
| S | 18 (42) | 6 (38) | 12 (44) |  |
| Cefotaxime, n (%) |  |  |  | 0.31 |
| R | 12 (28) | 6 (38) | 6 (22) |  |
| S | 31 (72) | 10 (63) | 21 (78) |  |
| Ceftazidime, n (%) |  |  |  | >0.99 |
| I | 1 (2.3) | 0 (0) | 1 (3.7) |  |
| R | 2 (4.7) | 1 (6.3) | 1 (3.7) |  |
| S | 40 (93) | 15 (94) | 25 (93) |  |
| Cefepime, n (%) |  |  |  | 0.58 |
| I | 2 (4.7) | 0 (0) | 2 (7.4) |  |
| R | 6 (14) | 3 (19) | 3 (11) |  |
| S | 35 (81) | 13 (81) | 22 (81) |  |
| Ampicillin Sulbactam, n (%) |  |  |  | 0.40 |
| I | 11 (30) | 6 (43) | 5 (22) |  |
| R | 5 (14) | 1 (7.1) | 4 (17) |  |
| S | 21 (57) | 7 (50) | 14 (61) |  |
| Unknown | 6 | 2 | 4 |  |
| Amoxicillin Clavulanic Acid, n (%) |  |  |  | >0.99 |
| I | 4 (9.3) | 1 (6.3) | 3 (11.1) |  |
| R | 2 (4.7) | 1 (6.3) | 1 (3.7) |  |
| S | 37 (86) | 14 (87.4) | 23(85.2) |  |
| Piperacillin Tazobactam, n (%) |  |  |  |  |
| S | 43 (100) | 16 (100) | 27 (100) |  |
| Aztreonam, n (%) |  |  |  | 0.32 |
| I | 3 (7.0) | 2 (13) | 1 (3.7) |  |
| R | 6 (14) | 1 (6.3) | 5 (19) |  |
| S | 34 (79) | 13 (81) | 21 (78) |  |
| Imipenem, n (%) |  |  |  |  |
| S | 43 (100) | 16 (100) | 27 (100) |  |
| Meropenem, n (%) |  |  |  |  |
| S | 43 (100) | 16 (100) | 27 (100) |  |
| Ciprofloxacin, n (%) |  |  |  | 0.89 |
| I | 4 (9.5) | 1 (6.7) | 3 (11) |  |
| R | 10 (24) | 3 (20) | 7 (26) |  |
| S | 28 (67) | 11 (73) | 17 (63) |  |
| Unknown | 1 | 1 | 0 |  |
| Levofloxacin, n (%) |  |  |  | >0.99 |
| R | 9 (21) | 3 (19) | 6 (22) |  |
| S | 34 (79) | 13 (81) | 21 (78) |  |
| Gentamicin, n (%) |  |  |  | 0.31 |
| R | 12 (28) | 6 (38) | 6 (22) |  |
| S | 31 (72) | 10 (63) | 21 (78) |  |
| Amikacin, n (%) |  |  |  |  |
| S | 43 (100) | 16 (100) | 27 (100) |  |
| Compound Sulfamethoxazole, n(%) |  |  |  | 0.50 |
| R | 19 (46) | 8 (53) | 11 (42) |  |
| S | 22 (54) | 7 (47) | 15 (58) |  |
| Unknown | 2 | 1 | 1 |  |
| Tetracycline, n (%) |  |  |  | 0.55 |
| R | 24 (56) | 8 (50) | 16 (59) |  |
| S | 19 (44) | 8 (50) | 11 (41) |  |
| Minocycline, n (%) |  |  |  | 0.50 |
| I | 3 (12) | 1 (20) | 2 (10) |  |
| S | 22 (88) | 4 (80) | 18 (90) |  |
| Unknown | 18 | 11 | 7 |  |
| Chloramphenicol, n (%) |  |  |  | 0.70 |
| R | 9 (21) | 4 (27) | 5 (19) |  |
| S | 33 (79) | 11 (73) | 22 (81) |  |
| Unknown | 1 | 1 | 0 |  |
