# Supplementary material for: Molecular determinants of Escherichia coli causing neonatal invasive infection following vertical transmission
Source: Front Cell Infect Microbiol. 2026 Jun 15;16:1855839. doi: 10.3389/fcimb.2026.1855839 (PMC13310911; doi:10.3389/fcimb.2026.1855839)
Supplement: Supplementary file 6 [file Table2.docx]

**Supplementary **Table 2. Sensitivity analyses comparing different prior distributions in Bayesian logistic regression models****

| **Characteristic** | **Model** | **Mean** | **SD** | **10% CrI** | **50% CrI (Median)** | **90% CrI** |
| --- | --- | --- | --- | --- | --- | --- |
| Intercept | Regularized | -1.3 | 0.7 | -2.2 | -1.2 | -0.4 |
|  | t-distribution | -2.1 | 1.0 | -3.3 | -2.0 | -0.8 |
|  | Wider prior | -2.3 | 1.1 | -3.7 | -2.2 | -1.0 |
| ***neuA*** | Regularized | 1.2 | 0.6 | 0.4 | 1.2 | 2.0 |
|  | t-distribution | 1.6 | 0.9 | 0.5 | 1.6 | 2.9 |
|  | Wider prior | 1.7 | 1.0 | 0.5 | 1.7 | 3.0 |
| **ST_95** | Regularized | 0.8 | 0.8 | -0.3 | 0.7 | 1.8 |
|  | t-distribution | 2.1 | 2.1 | -0.2 | 1.8 | 4.8 |
|  | Wider prior | 2.8 | 2.5 | -0.2 | 2.5 | 6.2 |
| ***iutA*** | Regularized | 0.9 | 0.6 | 0.1 | 0.9 | 1.7 |
|  | t-distribution | 1.2 | 0.8 | 0.2 | 1.2 | 2.2 |
|  | Wider prior | 1.3 | 0.8 | 0.2 | 1.3 | 2.3 |
| ***kpsMT II*** | Regularized | 0.9 | 0.7 | 0.1 | 0.9 | 1.8 |
|  | t-distribution | 1.5 | 1.0 | 0.3 | 1.5 | 2.8 |
|  | Wider prior | 1.7 | 1.1 | 0.4 | 1.7 | 3.1 |

Note: CrI = Credible Interval; The Regularized model employed regularized priors, the t-distribution model employed t-distribution priors, and the Wider prior model employed broader prior distributions. All parameter estimates are presented on the log-odds ratio scale. This table demonstrates the robustness of key predictors under varying prior specifications.
