# Supplementary material for: Molecular determinants of Escherichia coli causing neonatal invasive infection following vertical transmission
Source: Front Cell Infect Microbiol. 2026 Jun 15;16:1855839. doi: 10.3389/fcimb.2026.1855839 (PMC13310911; doi:10.3389/fcimb.2026.1855839)
Supplement: Supplementary file 7 [file Table3.docx]

****Supplementary Table 3. Evaluation of Interaction Effects Based on Updated LOO Comparison (4 Bayesian Logistic Regression Models)****

| Interaction Term Included | Interaction Coefficient Estimate | Interaction 95% CrI | LOO elpd_diff (SE) |
| --- | --- | --- | --- |
| **ST95 × *neuA*** | 0.60 | -1.10, 2.37 | **0.0 (0.0)** |
| **ST95 × *iutA*** | 0.60 | -1.10, 2.37 | **0.0 (0.0)** |
| **ST95 × *kpsMT II*** | 0.60 | -1.10, 2.37 | **0.0 (0.0)** |
| **None** | -- | -- | **-0.1 (0.1)** |
