# Supplementary material for: Molecular determinants of Escherichia coli causing neonatal invasive infection following vertical transmission
Source: Front Cell Infect Microbiol. 2026 Jun 15;16:1855839. doi: 10.3389/fcimb.2026.1855839 (PMC13310911; doi:10.3389/fcimb.2026.1855839)
Supplement: Supplementary file 8 [file Table4.docx]

**Supplementary Table 4. E-value analysis for key predictors**

| **Characteristics** | **OR** | **OR 95%CrI** | **E-value** | **E-value 95%CI** | **Probability of E-value≥1.5** | **Probability of E-value≥2.0** |
| --- | --- | --- | --- | --- | --- | --- |
| *neuA* | 4.95 | 1.65–16.44 | 9.73 | 1 - 70.38 | 96.1% | 93.8% |
| ST_95 | 6.05 | 0.82–66.7 | 11.19 | 1 - 599.29 | 84.8% | 81.4% |
| *iutA* | 3.32 | 1.22–9.03 | 6.17 | 1 - 32.67 | 92.3% | 88.4% |
| *kpsMT II* | 4.48 | 1.35–16.44 | 8.40 | 1 - 67.37 | 93.9% | 91.1% |
