# Supplementary material for: Molecular determinants of Escherichia coli causing neonatal invasive infection following vertical transmission
Source: Front Cell Infect Microbiol. 2026 Jun 15;16:1855839. doi: 10.3389/fcimb.2026.1855839 (PMC13310911; doi:10.3389/fcimb.2026.1855839)
Supplement: Supplementary file 9 [file Table5.docx]

| Variable | Original_p_value | Significant_original | FDR_adjusted_P | Significant_FDR |
| --- | --- | --- | --- | --- |
| *neuA*, n (%) | 0.0013 | TRUE | 0.0104 | TRUE |
| *neuS*, n (%) | 0.0027 | TRUE | 0.0108 | TRUE |
| *iutA*, n (%) | 0.0083 | TRUE | 0.0221 | TRUE |
| ST95, n (%) | 0.018 | TRUE | 0.036 | TRUE |
| *kpsMT II*, n (%) | 0.037 | TRUE | 0.0592 | FALSE |
| *chuA*, n (%) | 0.056 | FALSE | 0.0747 | FALSE |
| H7, n (%) | 0.069 | FALSE | 0.0789 | FALSE |
| *papA*, n (%) | 0.092 | FALSE | 0.092 | FALSE |

Supplementary Table 5. Variables with original p-values < 0.1 and the results of FDR correction

Note: FDR correction using the Benjamini-Hochberg method.
